# Clinician–researchers and custodians of scarce resources: a qualitative study of health professionals’ views on barriers to the involvement of teenagers and young adults in cancer trials

**DOI:** 10.1186/s13063-019-3942-y

**Published:** 2020-01-10

**Authors:** Ruth I. Hart, Nina Hallowell, Jeni Harden, Angela B. Jesudason, Julia Lawton

**Affiliations:** 1 Usher Institute, University of Edinburgh, Edinburgh, EH8 9AG UK; 2 Wellcome Centre for Ethics and Humanities and Ethox Centre, Nuffield Department of Population Health, Big Data Institute, Li Ka Shing Centre for Health Information and Discovery, University of Oxford, Oxford, OX3 7LF UK; 3 Royal Hospital for Sick Children, Department of Paediatric Haematology and Oncology, Sciennes Road, Edinburgh, EH9 1LF UK

**Keywords:** Trials, Equipoise, Role conflict, Teenagers & young adults, Cancer, Qualitative research, Interviews

## Abstract

**Background:**

Equipoise and role conflict have been previously identified as important factors in professionals’ engagement with trials, inducing behaviours which can impact on recruitment. We explored these phenomena as potential explanations for the low levels of involvement of teenagers and young adults (TYA) with cancer in clinical trials in oncology.

**Methods:**

We report findings from interviews with 30 purposively sampled direct-care professionals involved in delivering cancer care and/or facilitating clinical trials in Scotland. We undertook qualitative descriptive analysis, focussed on identifying key issues and themes.

**Results:**

Interviewees largely identified as clinician–researchers and portrayed oncology as a specialty in which research was integral to care. They saw their primary responsibility as ensuring patients received the best treatment, but asserted that, in general, trials provided a vehicle for optimal care. Role conflict in its traditional form was rarely evident; however, other tensions were manifest. Professionals found the significant time costs of delivering trials difficult to reconcile with the increasing pressures on clinical services. They felt a responsibility to make prudent choices about the trials with which to engage. Guided by utilitarian principles, these choices were oriented towards benefiting the largest number of patients. This favoured trials in high volume diseases; as TYA tend to have rarer forms of cancer, professionals’ support for—and TYA’s access to—relevant trials was, by default, more limited.

**Conclusions:**

Neither lack of individual equipoise nor experiences of traditional forms of role conflict accounted for the low levels of involvement of TYA with cancer in clinical trials. However, prominent tensions around the management of scarce resources provided an alternative explanation for TYA’s limited access to cancer trials. The prevailing approach to decision-making about whether and which trials to support was recognised as contributing to inequalities in access and care. Professionals’ choices, however, were made in the context of scarcity, and structured by incentives and sanctions understood by them as signalling governmental priorities. A franker discussion of the extent and distribution of the costs and benefits of trials work is needed, for change to be achieved.

## Background

Clinical trials have played a definitive role in medical progress, particularly in the field of cancer care [1]. Important patient outcomes, including survival, have improved significantly in that specialty since the expansion of trial activity in the mid-twentieth century [2]. However, not all cancer patients have had the same level of involvement in trials, and patient populations with lower levels of trial involvement have seen more modest improvements in outcomes. Teenagers and young adults (TYA) with cancer are one such group; levels of trial participation and improvements in outcomes for this group contrast particularly dramatically with those achieved in paediatric oncology, where trial participation levels have been high [3–5].

Whilst clinical trials are powerful tools for advancing knowledge and patient care, their successful delivery is difficult, with recruitment challenges affecting timely completion of a significant proportion of studies [6, 7]. A growing body of research has sought to understand the barriers and facilitators to recruiting into clinical trials, initially by exploring patients’ views and experiences [8–13]. More recently, researchers have investigated the perspectives of health professionals charged with recruiting, consenting, and retaining patient participants [14–17]. Two distinct but related explanatory concepts have been suggested as affecting professional recruitment practices and trial outcomes: the existence (or not) of equipoise and experiences of role conflict.

Clinical equipoise [18] is considered a fundamental ethical requirement of any trial involving random allocation. In essence, being ‘in equipoise’ means being in a state of uncertainty about the relative scientific or clinical merits of the different arms of a clinical trial. When there is uncertainty about which treatment is preferable, then conducting a trial is justified. Distinctions have, however, been drawn between clinical and individual equipoise (uncertainty), and these may not be fully aligned [15, 19]. So, while health professionals may agree that clinical equipoise exists, i.e. recognise that a lack of consensus exists amongst medical experts, equipoise at the individual level is often less stable. Individual healthcare professionals may believe that one form of treatment is generally superior, or that specific (groups of) patients would benefit more from one treatment than another. Disturbances in individual equipoise may lead professionals to undertake selective or biased recruitment, such as not broaching trial participation with certain groups or individuals, or introducing a trial in a way that prompts patients to decline involvement [14, 19–21].

Underpinning these behaviours is a concern that participation would not be in patients’ best interests. The care obligations at the heart of the clinician-patient relationship are deeply felt, and whilst clinical equipoise may provide ethical justification for allocation of patients to treatment according to a trial protocol, individual uncertainty will almost inevitably leave clinicians feeling conflicted. Broader tensions between goals and responsibilities relating on the one hand to research or scientific endeavours, most particularly clinical trials, and on the other to the care of (individual) patients, have been conceptualised as manifestations of role conflict [22–24]. Role conflict arises because clinical trials are driven by hypotheses, which are operationalised in protocols that determine the care a patient receives (in lieu of assessments of patients’ individual circumstances, preferences or needs). This can lead professionals to question whether the interests of their patients might not be better served by more tailored care, albeit that the provision of such care may not be consistent with the priorities of the trial. This sort of conflict has been identified as generating significant discomfort for health professionals in a range of positions and specialisms. As with lack of equipoise, where responsibilities to individual patients are deemed to conflict with and override those due to research endeavours, behaviours which may impact negatively upon recruitment can be triggered [20, 25, 26].

As part of a wider study of barriers (and facilitators) to TYA’s involvement in cancer trials, we interviewed health professionals involved in delivering cancer care and/or facilitating clinical trials. In keeping with the literature, we anticipated that a lack of equipoise (individual uncertainty) and/or role conflict might offer explanatory vehicles for the low levels of involvement of TYA in cancer trials. Our findings did not support this. In particular, they (our findings) did not show role conflict, as currently understood and employed in the literature, to account for limited trial participation by TYA. However, as we explain below, they revealed other new and highly significant tensions, with ultimately similar consequences for TYA’s involvement in trials.

## Methods

We outline our methods—and findings—broadly in accordance with the consolidated criteria for reporting qualitative studies (COREQ) [27]. The use of such checklists is advocated in and by many clinical and scientific journals [28], although it has been problematized by some prominent researchers [29].

### Design and theoretical framework

We undertook a qualitative study, involving one-off semi-structured interviews with direct care and other professionals, patients and family caregivers (data from non-care professionals, patient and caregiver interviews will be reported separately in due course). The study was characterised by an emergent, inductive design, purposive sampling and an iterative approach to data collection and analysis [30]. This iterative approach enabled us to revise our sampling strategy and interview topics, and to capture and unpack emerging issues (including some unanticipated when the study began). Our work was lightly informed by Normalization Process Theory (NPT), a middle-range theory offering an explanatory framework for ‘how complex practices… are made workable… in context-dependent ways’ (p.536) [31]. Amongst other things, NPT sensitised us to the potential for differing institutional contexts to shape and produce variation in interviewees’ experiences and perspectives, and the need to allow for this possibility in our sampling and interview questions.

### Setting

We conducted our study in Scotland. Health is a devolved matter, with legislation relevant to the National Health Service (NHS) in Scotland made by the Scottish Parliament. NHS Scotland is legally and financially independent from NHS England and has a distinct organisational framework. Fourteen regional ‘Health Boards’ plan, commission and deliver services, in line with the Scottish Government’s priorities. Major and specialist facilities are concentrated in the four largest health boards, although more than 20 hospitals in Scotland provide cancer care. Research infrastructure is provided by NHS Research Scotland (NRS), which takes its strategic direction from the Chief Scientist Office (CSO), an arm of the Scottish Government (and the funder of this research), and the four largest health boards.

### Participants and recruitment

Our approach to sampling was purposive and focussed on construction of a sample reflecting the diversity of relevant experiences and perspectives. Hence, we set out to recruit medical and nursing staff from different sub-specialties, types of service, hospitals and health boards (for example, small, large, rural, urban, with and without specialist TYA services/facilities). In light of the issues emerging in the first phase of the interviews, which related substantially to what might be termed structural factors (obstacles to the timely approval and set-up of relevant trials), we later sought to recruit professionals whose roles gave them particular insight into those matters. Initially, potential research participants were identified by clinical members of the research team. As sampling progressed, help to identify professionals with particular characteristics and/or experiences was sought from advisory group members and other clinical colleagues. We also invited interviewees to suggest colleagues with whom we might usefully speak. Interviewees were not asked to introduce us to their colleagues and were not informed whether their suggestions were followed up.

Approaches to potential interviewees were made in writing, either by the Chief Investigator (AJ) or the project researcher (RH), and were followed up by the latter. All potential interviewees were given detailed participant information sheets and invited to opt-in by replying directly to the project researcher. Approximately half of those approached agreed to take part. Where reasons were given for declining participation, these typically related to pressures of work and/or the small number of TYA patients seen. We discontinued recruitment once the team was in agreement that sampling ambitions had been satisfied and data saturation had been reached (i.e. the data set included an appropriate range of participants and perspectives, and additional interviews were no longer generating new issues or themes).

### Data collection

Data was collected between December 2017 and August 2018. Interviewees were given the option of either a face-to-face or telephone interview, with the former (16/30) typically taking place in the interviewee’s own office or a private meeting room. These were conducted by the project researcher (RH), a social scientist with prior experience of undertaking qualitative health research in haemato-oncology and other clinical areas.

Interviews were semi-structured, loosely following a topic guide outlined in Table 1. This was informed by a review of the literature, the expertise of the research team, and input from advisory group members. In line with study’s inductive and iterative approach, the topic guide was revised over the course of the study in light of emerging findings. Questions were modified in situ to take account of interviewees’ varying roles, experiences and perspectives. As such, the *guide* should be considered precisely that, and not as a script. Interviews typically lasted 45–60 min. All were audio-recorded and then transcribed verbatim. After anonymization had been fully confirmed, transcripts were imported into the specialist data management software, NVivo (Version) 11 (QSR International Pty Ltd., Doncaster, Victoria, Australia).

**Table 1:** Interview topic guide

About the interviewee:	
Role/responsibilities, including clinical and other interests	
Contact with TYA patients	
Experiences of providing care to TYA	
If/how providing care to older adults (or children) is different	
Involvement with trials:	
Understanding of the term	
Experience of trials work	
Reasons for doing trials	
Views on costs and benefits of trials (to current and future patients, professionals, and organisations)	
Decision-making about whether to open any specific trial locally	
Processes for opening a trial locally	
Barriers and facilitators to timely opening of trials	
Scope for improving processes	
Recruiting TYA (and other) patients to trials open locally	
Experience of recruiting TYA (and other) patients	
Processes for identifying eligible patients	
Deciding whether or not to approach individual patients	
Who/what is involved in recruitment	
Issues, concerns, and/or challenges specific to TYA (in general and/or sub-groups)	
Referring TYA (and other) patients to trials open elsewhere	
Experience of referring TYA (and other) patients	
Processes for identifying eligible patients	
Deciding whether or not to approach individual patients	
Who/what is involved in referral	
Issues, concerns and/or challenges associated with referral to trials open elsewhere	
Issues, concerns and/or challenges specific to referring TYA to trials open elsewhere	
Increasing TYA participation in trials	
Explanations for variation (in levels of participation) between services and sub-specialisms	
How to promote trial participation by this age group	
Other issues or comments	
Changes in context and/or trials	
Emergent issues	

### Data analysis

We undertook qualitative descriptive analysis, with a focus on producing rich, ‘minimally theorized’ descriptions of views and experiences [30, 32]. After initial familiarisation with the transcripts, which involved close reading and line-by-line coding, two members of the research team (RH and JL) undertook more focussed coding and ‘constant comparison’ [33]. Having undertaken independent analyses of the data, these researchers met to discuss and reflect upon their interpretations. The two analyses were very similar; hence, consensus on the coding frame for subsequent work was easily reached. The final stage of the analysis involved a further round of coding and constant comparison, with the project researcher (RH) systematically comparing, sorting and associating data and codes, until patterns, subtle variations and exceptions became clear. Summaries, diagrams and analytical reports enabled the analysis and gave other members of the team access to the data and the analytical logic.

## Results

We interviewed 30 direct care professionals whose role(s) involved delivering cancer care and/or facilitating clinical trials in Scotland. Participant characteristics are detailed further in Table 2.

**Table 2:** Participant characteristics

Interviewees with direct care responsibilities		30
Type of healthcare professional	Consultant	23
	Nursing	4
	Other	3
Consultants’ specialisms	Clinical oncology	7
	Medical oncology	6
	Haematology	6
	Paediatric medicine	3
	Surgery	1
Consultants’ site/sub-specialisms*	Haematological malignancies	5
	Gastro-intestinal cancers	4
	CNS/Neurological	3
	Genito-urinary cancers	3
	Breast cancer	2
	Gynaecological cancers	2
	Head, neck & thyroid cancers	2
	Sarcomas	2
	Paediatric oncology	2
	Lung cancer	1
	Melanoma	1
	MCUP	1
If also had research development or support responsibilities	No	25
	Yes	5
Type of responsibilities	Senior role in a CTU or CRF	3
	Specialist trial support**	2
Board to which primarily affiliated	NHS Lothian	11
	NHS Greater Glasgow & Clyde	8
	NHS Highland	4
	NHS Tayside	3
	NHS Grampian	2
	NHS Fife	1
	Other primary affiliation***	1

* Several consultants worked in more than one sub-specialism; therefore, these numbers do not sum to 23

** With regard to pharmaceutical or radiographic components

*** Interviewee primarily affiliated to an institution outside Scotland

In the sections that follow, we report how (1) these professionals largely identified as clinician–researchers and offered little evidence of experiencing role conflict in its traditional form, but (2) other powerful tensions had emerged around the use of scarce resources. Quotes are attributed to participants who are distinguished using unique alphanumeric codes, the initial letters of which provide information on their role: DC-C/N/O (Direct Care-Consultant/Nursing/Other). No relevant differences in perspective by role/staff group emerged.

### Identifying as clinician–researchers

Interviewees with direct care roles strongly identified as clinician–researchers, with accounts emphasizing the close integration between research and care in contemporary oncology. They portrayed involvement in research, particularly clinical trials, as an integral part of their work; something which was the norm, rather than the exception, for professionals within their specialism:

‘it’s part of core work… you can’t do oncology without doing research, it’s just… part of our job… just about every oncologist is involved in clinical trials… and if you’re not, you’ve got the question, why not?’ (DC-C-11).

‘in oncology, (research) is integral to clinical care… that’s what makes it such an interesting speciality, everyone’s involved… (and) it’s very integrated into clinical practice.’ (DC-C-12).

Clinicians described being introduced to clinical trials work early in their medical career and talked of taking on more responsibilities for trials work as they gained experience and status in oncology. Current oncology trainees, they said, were being prepared to take on similar responsibilities in the future. For example, as part of the medical oncology curriculum, trainees were all expected to undertake ‘GCP’ (Introduction to Good Clinical Practice) training, and to be involved in recruiting patients to trials, as well as providing follow-up care.

Some nursing interviewees were in research/trials nurse roles at the time of interview; others were not, but had held such posts in the past. Several recalled involvement, as staff nurses, in the care of cancer patients treated through clinical trials. This, they said, had given them too a strong sense of the importance of trials work and its contribution to improving cancer care:

‘you can see the development of drugs over the course of the years… you can actually see that with, you know, with your own eyes… that’s motivational, you know, to being positive about trials, and taking part in them.’ (DC-N-10).

Both clinicians’ and nurses’ accounts gave a strong impression of a trial culture, underpinned by a commitment to evidence-based medicine and with a focus on improvement, patient safety and the provision of the highest quality of care:

‘if we don’t do clinical trials, we will make decisions… not based on the best evidence… we have to do clinical trials to actually improve outcomes, but also to protect patients from drugs or interventions that might actually not be as effective as what’s already available.’ (DC-C-26).

Significantly, clinical trials were viewed not only as delivering benefits for future patients, but also as profiting current patients. Interviewees largely shared the opinion that research-active organisations achieved better results for patients (generally) and that patients who took part in trials had superior outcomes to those who did not. Interviewees acknowledged some uncertainty as to why patients in centres that delivered trials did better. They offered explanations concerning, inter alia, the culture or outlook developed by involvement in trials, and the value of external scrutiny of practice:

‘Trials make people think… (be) adaptable and willing to change for the better… that’s a culture that… taking part in clinical trials develops, or encourages.’ (DC-N-10).

‘we also know that… being part of research is quite good from a quality control point of view… your patient care… is being compared by the trials monitoring committee, it’s almost like… intellectual peer review… of practice.’ (DC-C-16).

A significant proportion of interviewees referred to ‘evidence’ suggesting that patients enrolled in trials had better outcomes. Again, interviewees acknowledged it was not yet entirely clear why trial-enrolled patients did better. They stressed that new treatments or new ways of administering treatment did not always outperform standard care and cited ‘research’ showing that control arm patients also tended to have better outcomes than non-enrolled peers:

‘there’s… evidence… that patients who participate in trials do better, irrespective of whether they’ve got the trial treatment.’ (DC-C-13).

Interviewees suggested that the explanation was probably ‘multi-factorial’ (DC-C-11), identifying a series of factors or mechanisms for what is sometimes referred to in the literature as ‘the trial effect’ [34]. These related largely to care practices—careful observation of a treatment protocol, scrupulous monitoring, and diligent recording:

‘there’s quite a lot of evidence to say that patients in clinical trials get better clinical care, just ‘cause they are seen more frequently, and so you’re much more likely to pick up on symptoms, and problems, at an earlier stage. So for individuals, it’s always important to stress that the benefit’s not necessarily from the drug, or the technique, because we don’t know yet if that’s going to make any difference, but we know that generally speaking, because of the more intensive care provided for people in trials for collecting all the data, that translates into sort of better clinical care.’ (DC-C-5).

Whatever the explanation, treatment through a trial was, in principle, viewed as giving patients access to the best possible care. As such, our interviewees gave little impression of being conflicted in the performance of their clinical care and research responsibilities; in contrast, they saw trials as a vehicle through which they could offer their patients optimal clinical care.

Accounting further for the apparent lack of conflict between care and research, interviewees reflected on the characteristics of the trials with which they were involved, and the distinctive nature of those of most relevance to TYA patients. Although some direct care interviewees reported involvement in early phase trials, most interviewees and accounts focussed on ‘Phase 3’ (large-scale, randomised) trials of first-line care. These are the sorts of trials involving the largest number of patients and, archetypally, compare two quite distinct drugs, procedures or technologies, one of which is new. The trials described by our interviewees appeared notable for their complexity, comparing differences in protocols which could involve multiple treatment modalities. Interviewees acknowledged that, *in principle,* such a trial might still expose patients to a new agent that was inferior to current care. However, several interviewees took pains to note that ‘new’ did not mean entirely unknown, one commenting as follows:

‘in (this sort of) trial you feel you’ve come far down the road enough, for, you know, the “new” drug not to be a worse drug. It (may) not be better, but there are reasonable data to say… it shouldn’t be worse.’ (DC-C-7).

They stressed that patient safety was the prime consideration in the design of trials and that all were very carefully scrutinised before initiation. Though laborious to navigate, regulatory and review processes functioned both to minimise harm and to reassure clinicians that they were not exposing their patients to inappropriate risks:

‘Our process for taking on trials, and the way that our consultants work… we’re quite confident that we’re not offering things to people that wouldn’t be good for them.’ (DC-N-10).

Moreover, interviewees described trials in the disease areas of most relevance to TYA patients (for example, sarcomas, brain tumours, and acute leukaemia) as commonly exploring different ways of using familiar treatments, sometimes using ‘different treatment approaches using the same drug’ (DC-C-9), rather than investigating the effects of new ones:

‘Some of the studies… are actually looking at what other medicines we can take out, or lower the dose of, to cut down on side effects, so… trials are not necessarily about *new* treatment.’ (DC-C-17).

‘Trials (can) offer an opportunity to access medicines that we otherwise wouldn’t be able to… (But) a lot of our trials… are not like that though… a lot of our trials involve making minor modifications to… standard treatments.’ (DC-O-24).

As the quotes above suggest, interviewees portrayed these trials, which seldom had commercial funding, as often looking at quite subtle modifications to care, for example, making adjustments to treatment combinations, order, dosage and/or frequencies. Interviewees talked of attending to ‘the finer details of the treatment… trying to clarify aspects of that treatment’ (DC-C-3). One explained how they proceeded:

‘building on what our, our gold standard, or our best available therapy is, and we’re trying to tweak the treatment slightly to improve outcomes, or add in a new treatment, or change the doses, or change the scheduling of it… (to) further improve the treatment… to improve the outcomes.’ (DC-C-17).

Priorities varied between disease areas / tumour types, but outcomes of interest included both survival and (short and longer-term) toxicities. The latter was viewed as of particular significance to TYA patients:

‘in both haematology and breast cancer… we’ve got such good outcomes really, that it’s all about trying now to look at, how little do we need to do to get the same outcomes, to keep the quality of life? So there’s quite a lot of work about how much can we pull back… and still achieve the same results… The younger population, those people are hopefully going to be cured, and then live till they’re 80, so you don’t want them to live with the side effects of the chemotherapy or radiotherapy’ (DC-C-15).

Such trials were framed as exposing TYA patients to worthwhile benefits and quite limited risks:

‘I’m fairly enthusiastic about putting my acute leukaemia patients in trials… because… they get a fairly good deal in these trials and the risk-benefits… are pretty good.’ (DC-C-7).

Furthermore, the nature of these trials meant that the superiority of any particular arm was exceedingly difficult to pre-judge:

‘the basis for the trial is that we don’t know which one’s going to be better, or better tolerated… that’s the nature of a Phase 3 trial, we can’t run it unless we think there’s genuine uncertainly about which one is best. (And in these trials) that difference is much less apparent to anybody, even (those) running the trial. It’s less clear what, whether you would have been better off, if you had the other bit.’ (DC-C-3).

### New tensions emerging

Whilst interviewees expressed a deep commitment to research and saw it as an integral part of their responsibilities, most were employed primarily to deliver clinical services:

‘most people in, in oncology are NHS doctors… who believe in research, who do research, but whose first commitment is to the NHS service.’ (DC-C-11).

‘although they are interested in research, and involved, and want to... be involved, they have a clinical workload… real patients, and real people, that have to be dealt with.’ (DC-O-30).

Many talked of growing pressures on core services: clinician and nursing time, pharmacy, radiology/radiotherapy services and laboratory staff and facilities. Certain fields (solid tumour oncology), services (radiology), and health boards (rural) were suggested as facing particular or persistent challenges:

‘in adult solid tumour oncology, we’ve had a severe shortage of staff… across the UK. So workload has been a huge issue…’ (DC-C-25).

Interviewees from one health board reported longstanding difficulties with clinical staffing, with vacant consultant posts leaving them heavily reliant upon locums. Meanwhile, colleagues in another health board described their pharmacy and radiology services as struggling to deliver standard care. They relied on their larger neighbours for some work related to routine clinical services:

‘we… use (City) labs for a lot of things anyway… that’s standard… if there’s extra tests… that can be an issue.’ (DC-N-22).

These pressures made the costs associated with undertaking trials, in particular staff time, increasingly hard to absorb. Although trials were framed by some interviewees as a potentially useful source of income, they were more frequently construed as drawing critical resources away from routine care. This may in part be reflective of the source and levels of funding for the TYA-relevant trials around which conversations revolved. All current and recent trials of clear relevance to TYA with cancer which featured in interviews were non-commercial, i.e. funded by charities or through (European) grant programmes.

Notwithstanding their commitment to participating in such research, many interviewees talked at length of the significant monetary and other (e.g. in-kind) costs associated with establishing, opening and maintaining these trials: ‘trials are demanding in time, they’re demanding in resource…’ (DC-C-16). They drew attention to the substantial time costs associated with both opening and delivering a trial. These costs included trial administration, recruitment, delivery of complex trial interventions, data collection and reporting.

The demands associated with trial administration received particular attention and were framed both as significant and expanding. An experienced clinician remarked how, over their professional lifetime, they had ‘ballooned, absolutely ballooned’ (DC-C-25). Comments were also made that whilst non-commercial trials used to be more straightforward to administer, they were now expected to meet the same regulatory and reporting requirements as commercial trials—but without equivalent resources and infrastructure. Opening a trial was portrayed as extremely laborious and, critically, due to the requirement to maintain and update documentation to reflect (increasingly frequent) amendments, the ongoing demands on staff time were also considerable:

‘Seven or eight years ago, you would maybe see one or two protocol amendments in the lifetime of a study. Now you’re seeing two or three a year, it’s out of control, there’s more protocol amendments than patients on half our studies. And staying on top of that is extremely difficult.’ (DC-O-9).

Interviewees emphasised that, in large part, these administrative demands bore no relationship to the scale of recruitment to the trial. Due to broadly equivalent costs of activation, set-up and maintenance, trials where recruitment might be expected to be limited (often the case with those in disease areas relevant to TYA) could prove as much a drain on resources as those with much higher levels of recruitment:

‘if we do a trial where we’re going to recruit 10 patients, then obviously the amount of work that I have to do as a PI… and some of the nurses have to do – in terms of bureaucracy – is actually pretty much the same as if we were to do a trial where we might only recruit one.’ (DC-C-25).

Hence, in this context of high demand and scarce resources, accounts suggest that trials were in competition not only with clinical services, but also with each other. Interviewees drew attention to the ‘finite’ nature of resources, the ‘opportunity cost’ of trials work, and the consequential requirement to make prudent choices about *which* trials to engage with and fight for support for:

‘as a department, we’ve got limited research resource, so we… focus on trials… (with) a chance of benefiting our patients. Focus our nursing and data management resource into that.’ (DC-C-11).

Pressed to explain further how decisions were made whether to engage with (i.e. open) a particular trial or not, interviewees talked about assessing if a trial was ‘worthwhile’. This appeared to mean a range of different things, prominent amongst which was recruitment potential:

‘it’s about numbers, how many people do you need to get into a trial in order to make the trial… worth the resource…?’ (DC-C-3).

Professionals appeared to evaluate trial opportunities according to the principles of utilitarianism, which, at its simplest, is an economic philosophy oriented towards achieving the greatest good for the greatest number of people. Interviewees acknowledged that the prevailing value system privileged high volume diseases (for example, breast and prostate cancer). As one interviewee put it, ‘the big tumour types… dominate’ (DC-C-11). Accounts suggest that the system of rewards and penalties for trials work established by the Scottish Government (CSO) had encouraged and embedded such an approach. Its use of recruitment figures as the central metric incentivised trials in those centres, disease types, sub-types and phases where there was greatest potential to recruit:

‘Not to beat around the bush, we get very little money, until we recruit patients. You know, most of the incentivisation… bean-counting, is related to the numbers of recruits, not to the numbers of… studies. And so, you know, those are resources which, we’re putting in, where we may get nothing back, and would frankly be better directed to something where we’re gonna get something back.’ (DC-C-28).

Some interviewees questioned the rationale of the contemporary rewards system, musing that:

‘number of recruits to studies… that’s a slightly lazy way of trying to appreciate the quality and quantity of patient benefits… if you run a study on relatively few patients, but actually that’s a novel, good intervention, that would be a great benefit… the health impact may be… more significant that a study that runs on 100 patients which provides relatively minor, additional health benefits… Yet the 100-patient (study) would win, in the eyes of the Government.’ (DC-C-27).

Ultimately, and notwithstanding ambitions to have trials available for as many different patient groups as possible, there was widespread recognition that, in reality, only a minority of patients aged 16+ had access to care through a trial. One interviewee (DC-C-20) put it very bluntly: ‘for the majority of patients, there isn’t a trial.’ Significantly, however, the prevailing logic and rewards metric led to patterned inequalities in access to trials, powerfully dis-incentivising the opening of trials relating to rarer cancers such as those with which TYA typically presented:

‘(be)cause the numbers are small… staff are kind of dis-incentivised… A lot of people just say, I just can’t be bothered with that if I’m only gonna recruit, you know, one patient in six months or something, why the hell do I wanna do that? I’ve got other things to do.’ (DC-C-25).

Some interviewees highlighted a wider lack of trials in rare tumours, framing pharmaceutical companies as having limited interest in developing and trialling new treatments for such diseases (as the returns were likely to be modest and only distantly realised). Others questioned the extent to which smaller centres, outside London, were given an opportunity to participate where such trials were established. Many, however, explicitly acknowledged the role—and consequences—of local decision-making:

‘There is a national trial for rhabdos, but we haven’t opened it in, the adult site, because it’s a very complicated trial, and we would probably only get one patient every two or three years… ‘cause it only goes up to (age) 21, I think’ (DC-C-3).

‘there are some trials for very rare cancers… quite a few that are relevant to this group (TYA), where we have had to just make the decision, we don’t have enough resources to open this trial that we may recruit one, or zero patients (to) over the lifetime of the trial… that’s a big problem… for this group of patients.’ (DC-C-18).

It seems TYA patients might not be deprived of trial opportunities by their age per se, but by their tendency to present with tumours uncommon in the wider adult population:

‘we’ve got about 160 trials open… (but) there’s probably about half a dozen… which would include diseases which were relevant (to TYA).’ (DC-C-28).

## Discussion

A key finding of our research is that interviewees identified strongly as clinician–researchers and portrayed oncology as a specialty in which research was integral to care. Examples of role conflict in its traditional form [22–24] were largely absent from our data. However, there was evidence that new and significant tensions had emerged, which offered a powerful alternative explanation for the low levels of involvement of TYA with cancer in clinical trials in oncology. Specifically, our data suggest that, inasmuch as research was felt to conflict with care, it was in its potential to consume increasingly scarce and precious clinical resources. Our data reveal an acute appreciation of resource scarcity and a sense of obligation amongst professionals to make deeply pragmatic choices about those trials in which to invest their time. Guided by utilitarian principles, these choices were oriented towards benefiting the largest number of patients. This favoured trials in high volume diseases; as TYA tend to have rarer forms of cancer, their access to trials was, by default, limited. Interviewees recognised that the choices they made as professionals about which trials to support had very concrete repercussions for equality of access—and arguably care. TYA and other patients presenting with rare cancers (and indeed other rare diseases) were acknowledged as being systematically disadvantaged.

These findings do not fit entirely easily with the existing literature. Traditionally, the roles of clinician and researcher have been understood to be fundamentally distinct and inherently conflicting. Attention has been and continues to be drawn to differences in the goals, practices, responsibilities, obligations, risks and ethical frameworks for research and care [20, 22–24, 26]. The form and consequences of these role conflicts (for clinicians, patients and, to a lesser extent, researchers) have been relatively well documented, including in studies focussed on the conduct of clinical trials [14, 16, 35]. Role conflict has been found to have implications for both recruitment and trial delivery; for example, previous work has shown that clinicians will not follow a trial protocol if they are concerned that it will deprive their patients of the best clinical care [36].

That said, recent years have seen the publication of a growing body of work which challenges the conceptualisation of care and research as unerringly distinct and conflicting. That work suggests a more permeable boundary between research and care, at least in some technical specialities [37–39]. Keating and Cambrosio [1] and Cambrosio et al. [40] have argued that, in the field of oncology, the practices of standard care and participation in trials have become closely intertwined. Our own work very much supports that claim. Whilst interviewees were clearly cognisant of the clinical essence of their identity [20] and saw their primary responsibility as ensuring patients received the best clinical care, they also viewed trials, in general, as a vehicle for providing optimal care. This finding is in line with expert opinion [41] and other research [37], which similarly has found clinicians to perceive trial enrolment as a means to provide their patients with first-class care.

Interestingly, our data showed professionals to be overwhelmingly in (individual) equipoise. We surmise that the nature of TYA-relevant trials may be significant in this. Our data suggest that whilst TYA-relevant trials may involve new treatments, they frequently compare relatively similar regimens, involving differential delivery of familiar treatments. Where differences between treatment arms are subtle, it would seem less likely that professionals might hold strong beliefs regarding the likelihood of particular patients or patient groups benefiting more from one treatment (arm) than another. Arguably, in such cases, the potential for individual uncertainty about involving one’s patients in such a trial—and all the concerns and conflicts arising from or fuelled by this—would be reduced.

Although traditional forms of role conflict were largely absent from our data, powerful tensions, or conflicts, were prominent in relation to resource management. Highlighting a backdrop of fiscal crisis, or at least constraint, our interviewees portrayed themselves as having secondary, but nonetheless significant, roles as custodians of scarce resources. They indicated acute awareness of the finite nature of NHS resources and a sense of duty to use these to maximum effect. Many talked at some length of their struggles to reconcile the significant costs associated with involvement in trials with the increasing pressures on their clinical service. Other authors have documented the considerable time and other costs associated with involvement in trials [42–44], with some citing financial constraints as a significant barrier to involvement in (non-commercial) trials [37]. More than a decade ago Snowden et al. [45] argued that financial considerations shaped all aspects of trial work and deserved far closer scrutiny than they had received. Our study helps to fill important gaps in understanding of the actual—and perceived—economics of trials research, and how these mould professionals’ decisions and behaviours.

Our data highlight the significant and growing challenges for healthcare professionals of being involved in research—a product of the burgeoning pressure on clinical services and the increasing cost and complexity of clinical trials. These challenges meant that, notwithstanding a deep commitment to trials, only a minority of patients (aged 16+) could be offered treatment through a trial. Scholars working in other jurisdictions have similarly noted that, whilst central to the specialty, in practice, relatively few (adult) oncology patients take part in trials [1, 46]. Our interviewees emphasized their responsibility to make parsimonious choices between trial and other activities and portrayed trials as being in competition both with clinical services and each other. They described making deeply pragmatic decisions about trial engagement, with decisions being oriented towards containing costs and maximising returns. These appeared to follow a crudely utilitarian logic where benefits are calculated according to the number of potential recruits. We see parallels here with Lipsky’s depiction of the classic ‘street-level bureaucracy’, where demands on professionals always exceed the resources available, leading to the development of routines and rationing mechanisms enabling the maximal utilisation of resources whilst maintaining ‘a conception of… performance relatively consistent with ideal conceptions of the job’ (p.151) [47].

The current system of rewards and penalties for trial activity appeared to reinforce a focus on ‘high volume’ diseases and to explicitly dis-incentivise more complex or uncertain (in terms of recruitment) trials work. Again, this finding is consistent with Lipsky [47], who argues that discretionary behaviours may ‘add up’ to policy, but emerge in and from a context which not only structures the choices available, but also provides incentives and sanctions which, in turn, are correctly understood by workers as signalling leadership/management priorities. Such incentivisation schemes are both widespread in the NHS and widely problematized (see for example the debate around the Quality and Outcomes Framework (QOF) [48]). Professionals interviewed for this study recognised that their choices produced patterns—and, as a consequence, inequalities—in access to trials. Interviewees acknowledged that patient groups characterised by small numbers, such as TYA—who tend to present with rare types of cancer—and/or are served by smaller treatment centres, were amongst those most consistently disadvantaged. Differential treatment of different patient groups was, in line with Lipsky, rationalised as serving “the best interest of the greatest number’ (p.112) [47]. However, while choices were rationalised, their potential to explain and compound disparities in health outcomes acted as a source of some discomfort to interviewees.

Although the consequences of this new form of role conflict may be similar to those of more traditional forms, the causes are quite different. As such, proposed solutions to traditional forms of role conflict, such as, for example, more or better training on the concepts central to trials and how best to communicate these to patients [49], would appear to have uncertain relevance. Guidance not on the mechanics of recruitment but instead on how to balance competing obligations and undertake ‘boundary work’ [50] might be more valuable. Critically, more than a decade ago, Raftery et al. [51] called for better understanding of the organisational and resource aspects of trials and how these impact on research involvement and recruitment. This need does not as yet appear to have been fully met. Recognition of the increasing complexity and cost to health care institutions of hosting trials [41] would seem to us to be a necessary condition of change. Changes to current systems of rewards and penalties for trial activity may also be needed, if health professionals are to be encouraged to make different choices.

### Strengths and limitations

Qualitative research can improve understanding of the views, behaviours and decisions of health professionals (and their patients) [28, 52, 53]. It can also prompt the problematization and refinement of concepts and ideas, as demonstrated above. Critiques often concern generalisability; although such generalisability is rarely the goal of qualitative research, it is of interest whether, and with what caveats, findings have wider application. The nature of study samples is key to this. As noted in our Methods, sampling was purposive and continued until we were confident ‘saturation’ was achieved. Interviewees’ views were largely consistent, with strong patterns emerging during analysis. However, importantly, almost two-thirds of our interviewees came from the two largest Scottish health boards. Moreover, not all professionals approached agreed to take part in an interview. Though interviewees’ statements regarding their peers and the culture of their specialty suggest that accounts are reflective of a wider, prevailing view, this is not something of which we can be entirely sure. It is possible our sample is skewed towards more research-engaged clinicians; the perspective of those not approached or declining involvement might be different, with the latter perhaps not seeing our study as something to which they could usefully contribute. Also of note is that a relatively small number of nurses were interviewed. Though differences in perspective could not be discerned in our data, future research might usefully explore the perspectives of this staff group in more depth. Looking beyond oncology, Cambrosio et al. [40] advocate caution in extrapolating between specialties and indeed suggest that oncology may be distinctive. We have already highlighted a number of ways in which the trial work the interviewees were engaged in appeared idiosyncratic. We note here, however, that work on trials in other clinical areas, for example, diabetes, has identified similar concerns about financial pressures and difficulties undertaking less profitable studies [16]. Finally, as to whether our findings have relevance beyond the country and health service context in which the research was undertaken, this question hinges on the comparability of service organisation and resourcing. We would encourage readers with direct experience of other healthcare systems to think critically about and explore further the application of our findings to those contexts. Further research may be warranted.

## Conclusions

Accounts revealed growing tensions between the ethos of the clinician–researcher, who delivers the best care through the vehicle of trials, and the need to make pragmatic economic choices which inevitably diminish some patient groups’ access to such care. The utilitarian logic underpinning professionals’ decision-making about engagement in trials was recognised by some interviewees as having consequences for equality of care, in particular, placing patients such as TYA, who typically present with rare types of cancer, and/or who are served by smaller treatment centres, at a disadvantage. The consequences of inequality in access to trials were not explicitly explored in this study. However, denial of those benefits associated by interviewees with trial participation might reasonably be inferred. Moreover, prior research suggests that interviewees’ discomfort with the situation has strong foundations. For example, scrutiny of outcomes in TYA cancer patients has indicated that rates of clinical trial participation and improvements in survival (or lack of these) are closely associated [3, 4]. In the United States, ‘inclusion in research has come to be seen as an important strategy for reducing health disparities’ (p.338) [54], with recognition of inequalities in access to trials along lines of race and gender prompting legislation to promote the involvement of what were subsequently deemed ‘special populations’ [1]. We do not go so far as to suggest that such an approach be taken here, but argue that at the very least a far franker debate is needed if the issues our study highlights can begin to be resolved.

## Data Availability

The datasets (interview transcripts) reported on in this paper are not publicly available as rendering them entirely unidentifiable would require significant redaction, and the research participants did not consent to public data sharing.

## Abbreviations

NHS: National Health Service
NPT: Normalization Process Theory
PI: Principal Investigator
TYA: teenagers and young adults
